# Access to and adequacy of psychological services for adult patients in UK hospices: a national, cross-sectional survey

**DOI:** 10.1186/s12904-021-00724-3

**Published:** 2021-02-10

**Authors:** Daisy McInnerney, Bridget Candy, Patrick Stone, Nicola Atkin, Joana Johnson, Syd Hiskey, Nuriye Kupeli

**Affiliations:** 1 Wing B, Floor 6, Marie Curie Palliative Care Research Department, Division of Psychiatry, UCL, Maple House, 149 Tottenham Court Road, London, UK; 2 Parkville Integrated Palliative Care Service, Peter MacCallum Cancer Centre, Melbourne, Australia; 3 Marie Curie Hospice, Hampstead, London, UK; 4 The Oaks Hospital, Colchester, UK

**Keywords:** Hospices, Palliative care, Psychology, clinical, Mental health, Surveys and questionnaires

## Abstract

**Background:**

Providing psychological support to people living with terminal illness is a fundamental part of hospice care. Recent research on delivery of psychological services in hospices in the United Kingdom (UK) on a national level, including inequalities or variation in practice, is limited. A nationwide survey will highlight any differences in provision and in doing so help focus future research and inform best practice both within the UK, and internationally. The specific objectives of this survey are to (1) chart the types of psychological support available to adult patients in hospices in the UK in line with the National Institute for Health and Care Excellence model; (2) explore how services are organised; and (3) gather service perspectives on adequacy of care, and facilitators and barriers to appropriate practice.

**Methods:**

A cross-sectional online survey emailed to adult hospices in the UK in November–December 2019. One staff member involved in the delivery and/or organisation of psychological support was invited to participate per hospice. Of 193 invited hospices, 116 took part.

**Results:**

Sixteen percent rated their hospice psychological service as wholly adequate. The majority reported that services can access specialist professionals, but many relied on external referrals. Barriers to best practice included funding and staff capacity; facilitators included clear referral structures, audit and appropriate needs and outcome assessments.

**Conclusions:**

Access to psychological professionals has improved since the last survey 15 years ago, but the majority of responders felt their overall service was not wholly adequate. Basic emotional support is largely felt to be sufficient, but our results indicate a need for improvements in access to more specialist care. Partnerships with external mental health services may be key. Our findings highlight core facilitators and barriers to providing good psychological care at the end of life that should be considered by services both within the UK and on an international level.

**Supplementary Information:**

The online version contains supplementary material available at 10.1186/s12904-021-00724-3.

## Background

People living with terminal illness are likely to experience significant psychological distress [1–5]. Psychological and emotional support are a fundamental part of the holistic care provided by hospices. National and global health bodies, including the World Health Organisation and the European Society for Medical Oncology, recognise this as a priority area of focus for end-of-life care services [6–10]. Whilst its importance is widely acknowledged, relatively little is known about how psychological support is delivered in hospices both internationally and on a United Kingdom (UK)-wide scale.

The UK’s National Institute for Health and Care Excellence (NICE) last published detailed guidance on structuring psychological support in palliative care for adults in 2004 and only in cancer [11]. These guidelines propose a four-level model, designed to ensure that all patients are psychologically assessed and have access to appropriate psychological support at key points during their illness (such as at diagnosis, starting treatment or when the disease becomes incurable). Level 1 care (providing compassion and information) should be provided by all health and social care professionals. Levels 2 to 4 require staff with specialist training. This ranges from health professionals with additional mental health expertise at Level 2 (such as general practitioners, social workers, nurse specialists and some complementary therapists), through to trained accredited mental health professionals at Level 3 (such as counsellors and psychological therapists), and mental health specialists at Level 4 (such as psychiatrists and clinical psychologists). At present it is unclear how widely this structure has been implemented across UK hospices. In 2019, NICE published service provision guidelines for end-of-life care highlighting that health and social care practitioners should have the skills needed to provide psychological support [12]. However, these guidelines do not describe what skills are required, how they should be developed, or how the support should be structured or delivered.

There has not been much recent national research on psychological services at the end of life, but available evidence suggests that provision may be limited. The last UK-wide survey of psychological services within hospices, conducted in 2005, found less than half (41%) of UK hospices had access to a clinical psychologist [13]. The survey also found hospices often relied on referral to external mental health services to provide specialist psychological and psychiatric support to their patients. Despite this, 41% reported problems accessing services provided by local mental health trusts. Another more recent survey of palliative care physicians working in hospice and hospital settings in the UK found provision of psychological support remained limited, with the majority of respondents (64%) reporting difficulty accessing psychological services [14]. This survey highlighted lack of formal referral systems as one of the main challenges to accessing these services. In a 2018 survey, a third of clinical psychologists working in UK hospices rated their input as ‘not at all sufficient’, although the sample size was small, with just 18 responses [15]. Of these, more than two-thirds of respondents were employed by NHS trusts, rather than directly by the hospice, emphasising again a clear role for partnerships and referrals to services outside of the hospice itself.

It is important to have a more up-to-date understanding of the current delivery of psychological services in UK hospices, which can in turn be used for regional and global comparisons. This survey aims to identify patterns and variation in psychological service delivery across the UK including examples of, and barriers to, good practice. This may help inform efforts to ensure there is equality in service provision, both by identifying areas of disparity, and ways of improving care. To our knowledge, we are unaware of any equivalent studies in the UK or elsewhere in the world. Since the UK has one of the most extensive palliative care services worldwide [16], the findings may well provide useful insights for services in other countries regarding best practice and barriers to achieving it. Finally, it is important to note that this survey was conducted before the COVID-19 pandemic, which has resulted in fundamental changes to the way hospices in the UK operate [17]. The long-term effects of the pandemic on the ways in which care are delivered remain to be seen at the time of writing. However, the results of this survey can act as a benchmark to compare psychological support before, during and after the pandemic.

## Methods

### Aims

The aims of this survey are to:

1. Collect information on the types of psychological support that are available to adult patients in UK hospices and who delivers them.
2. Increase understanding of how psychological support services are currently organised within UK hospices.
3. Explore views of UK hospice staff involved in delivery of psychological services on adequacy of current psychological care being offered to patients, and any facilitators of and barriers to effective delivery.

The survey also explored psychological care for family carers. This will be reported in a separate paper.

### Design

A survey using online platform Opinio v7.11 [18] to collect quantitative and qualitative data, open for six weeks in November to December 2019. This paper is reported in line with the Checklist for Reporting Results of Internet E-Surveys (CHERRIES) guidelines [19] (Additional File 1).

### Setting and participants

The survey was emailed to 193 of 195 hospices providing care to adults in the UK on the Hospice UK list of service providers. Two National Health Service (NHS) hospices were not approached as they were not able to issue trust approval before the closing date. We aimed to collect one response per hospice.

#### Inclusion criteria

A member of the hospice team who was closely involved in the organisation and/or delivery of psychological services, and was well-positioned to comment on the services provided.

### Recruitment

Hospices were contacted by phone. We explained the aims and eligibility criteria. If we were able to speak to an appropriate member of staff at that time, their email address was requested and an invitation email sent (Additional File 2), linking to the participant information sheet and questionnaire. If a potential participant was not available at that time, depending on hospice preference, we either: (a) sent the invitation email to the reception email address to forward onto the appropriate person; (b) provided our contact details to pass onto the appropriate person; or (c) asked the best time to call back. If after calling three times we were unable to speak to an appropriate member of staff, the invitation email was sent directly to the most appropriate publicly available email address. Reminder emails were sent three, two and one week before the survey deadline. Awareness of the survey was promoted via the Hospice UK conference [20] and mailing list, and Marie Curie social media. All participants were offered a certificate of participation, and the option to enter a draw for one of two £30 vouchers.

### Screening

Screening was based on self-reported eligibility. The eligibility criteria were explained on the initial phone call, in the invitation email, information sheet and questionnaire. Participants completed an online written informed consent form confirming their eligibility before being able to progress to the questionnaire.

### Survey development and piloting

The open questionnaire was developed based on previous surveys, in discussion with a group of stakeholders, including a Patient and Public Involvement (PPI) representative, clinical psychologists and a palliative care consultant (Additional File 3) [13–15, 21, 22]. It contained 18 multi-part, multiple-choice questions and five open-response questions in four sections: (1) Basic information about respondents; (2) Organisation of psychological support; (3) Types of psychological support; (4) Access to psychological support. It was piloted for two weeks at nine hospices in October 2019 [20]. Three responses were received. Those who completed it reported the questionnaire was clear and easy to complete within the 15–20 min time estimate. Pilot responses are included in the final analysis.

### Data management and statistical methods

Complete and incomplete responses were included in the analysis. Complete (not completed) responses were defined as respondents who completed the final question of the survey. Duplicated responses were identified by checking email addresses of respondents during data cleaning, and only the most complete response included in the analysis. Quantitative responses were summarised using percentages generated in SPSS Version 24 [23]. Percentages were based on the number of respondents answering each question and rounded to the nearest whole number. Associations were deduced by eyeballing the data, but not tested using inferential statistics since hypotheses were not defined a priori*.* Free-text responses were analysed with inductive thematic analysis using QSR International Nvivo 11.4 software [24, 25]; the coding framework and themes were developed by one author (DM) and checked, discussed and refined with the research team.

### Ethics

The project was approved by the UCL Research Ethics Committee (ref: 15281/001) and the NHS Health Research Authority (HRA) (ref: 265276).

## Results

### Sample characteristics

The survey received 116 unique responses (response rate = 60%), of which 92 were complete. Table 1 reports sample characteristics. Ninety-eight (84%) responses were from hospices based in England, 11 (9%) in Scotland, 5 (4%) in Wales and 2 (2%) in Northern Ireland. The distribution of respondents across the four nations was similar to the distribution of Hospice UK list members (84, 8, 6 and 2% respectively). The ratio of NHS (5%) versus non-NHS (95%) managed services was also similar to the Hospice UK members list (7% NHS). One hundred (86%) of the hospices that responded provided a community care service in addition to inpatient beds and/or a day centre or outpatient clinics. This is in line with data which shows 83% of hospice care in the UK is provided in community-based settings [26].

**Table 1:** Characteristics of respondents and participating hospices

Characteristics		n (%) *n = 116*
Participant job title	Clinical services manager	**17 (14)**
	Chief executive	**1 (1)**
	Clinical psychologist	**13 (11)**
	Hospice manager	**4 (3)**
	Patient and family support services manager	**19 (16)**
	Psychological services manager	**11 (9)**
	Supportive care services manager	**6 (5)**
	Medical doctor	**7 (6)**
	Other doctor	**1 (1)**
	Other	**63 (54)**
Hospice region	England	**98 (84)**
	Northern Ireland	**2 (2)**
	Scotland	**11 (9)**
	Wales	**5 (4)**
Hospice management	An independent charity (even if partly funded by the NHS)	**109 (95)**
	An NHS hospice (even if partly funded by charity)	**7 (5)**
Inpatient beds at hospice	Yes	**100 (86)**
	*Median number of beds (IQR)*	*16 (9)*
	*Median number of admissions per week (IQR)*	*6 (3)*
Day centre at hospice	Yes	**104 (90)**
	*Median number of patients attending per week (IQR)*	40 (34)
General palliative care outpatient clinics	Yes	**83 (72)**
	*Median number of patients attending per week (IQR)*	*11.5 (22.5)*
Community palliative care team	Yes	**100 (86)**
	*Median number of patients on caseload (IQR)*	*120 (32)*
	*Median number of new referrals per week (IQR)*	*15 (22)*

Abbreviations: *IQR* Interquartile range

Respondents had a range of roles at the hospice where they work. The most commonly selected roles were Patient and Family Support Services Manager (16%), Clinical Services Manager (15%) or Clinical Psychologists (11%). 54% of respondents selected ‘Other’; within this category, exact job titles specified in free-text responses varied widely, with the most common being counselling, psychological wellbeing, patient or family support team leads (*n* = 19) and counsellors (*n* = 11).

### Psychological support services available to hospice patients

The types of psychological support professionals and volunteers available to hospice patients are shown in Table 2. The majority of hospices have access to an employed in-house complementary therapist (81%), spiritual advisor (78%) or counsellor (75%). 19% of hospices have in-house access to a clinical psychologist, and 9% to a counselling psychologist, while 44 and 49% respectively rely on referral to an external service to access these specialist professionals. There were no clear associations between type of care (i.e. inpatient, outpatient, day centre or community) and access to psychological support professionals, or between country and access to psychological support professionals. Table 3 shows the percentage of the hospice sample using each therapeutic approach. Hospices draw on a range of therapeutic approaches, most commonly: mindfulness strategies (91%), psychotherapeutic approaches (70%), Cognitive Behavioural Therapy (CBT; 68%) and art therapy (67%).

**Table 2:** Availability of psychological support professionals for adult patients in UK hospices, n (%)

Role	In-house		By referral		No access	Unknown
	Employee	Volunteer	Employee	Volunteer		
Spiritual advisor *(n* = 107)	83 (78)	64 (60)	19 (18)	13 (12)	1 (1)	0 (0)
Complementary therapist *(n* = 108)	87 (81)	70 (65)	3 (3)	6 (6)	3 (3)	0 (0)
Social worker *(n* = 101)	72 (71)	7 (7)	23 (23)	3 (3)	12 (12)	0 (0)
Creative therapist *(n* = 95)	54 (57)	34 (36)	5 (4)	9 (9)	14 (15)	2 (2)
Clinical psychologist *(n* = 82)	21 (19)	0 (0)	44 (41)	3 (3)	20 (19)	0 (0)
Counselling psychologist *(n* = 67)	6 (9)	6 (9)	28 (42)	5 (7)	26 (39)	1 (1)
Counsellor *(n* = 106)	80 (75)	58 (55)	15 (14)	8 (8)	5 (5)	0 (0)
Psychiatrist *(n* = 80)	3 (4)	0 (0)	62 (78)	3 (4)	16 (20)	0 (0)
Dual qualified professional *(n* = 68)	26 (38)	2 (3)	3 (4)	2 (3)	34 (50)	5 (7)
Registered Mental Health Nurse (n = 67)	5 (7)	2 (2)	36 (33)	3 (3)	21 (19)	2 (2)
Psychotherapist *(n* = 77)	32 (42)	10 (13)	21 (27)	3 (4)	20 (26)	2 (3)
Occupational therapist *(n* = 96)	79 (82)	5 (5)	16 (17)	1 (1)	5 (5)	1 (1)

‘No access’ refers to respondents that chose the multiple-choice option ‘No access’. ‘Unknown’ refers to respondents that chose the multiple-choice option ‘Unknown’. Respondents that did not select any answer for a therapist type were excluded from the analysis of that therapist type

The n number reported in the ‘Role’ column reports the number of responses received to the question

**Table 3:** Therapeutic approaches used by psychological support teams in UK hospices

Approach	Number of hospices using the approach, n (%)
Cognitive Behavioural Therapy (*n* = 91)	63 (68)
Acceptance and Commitment Therapy (*n* = 83)	40 (41)
Compassion Focused Therapy (*n* = 82)	42 (51)
Mindfulness Strategies (*n* = 102)	93 (91)
Narrative Therapy (*n* = 83)	41 (49)
Solution Focused Therapy (*n* = 84)	49 (58)
Systemic Therapy (*n* = 77)	37 (48)
Psychodynamic approaches (*n* = 83)	52 (63)
Psychotherapeutic approaches (*n* = 79)	55 (70)
Music therapy (*n* = 84)	32 (38)
Art therapy (*n* = 89)	60 (67)
Writing-based therapy (*n* = 78)	29 (37)
Hypnotherapy (*n* = 77)	21 (27)

The *n* number reported in the ‘Approach’ column reports the number of responses received to the question

### Organisation of available psychological services

The majority of respondents (57%) described themselves as wholly familiar with the NICE model of psychological assessment and support, 30% were mostly, 11% partly and 2% not at all familiar. There did not appear to be any association between level of familiarity with the model and the nation the hospice is based in. Psychological specialists (e.g. clinical psychologists, counsellors) appeared to be more likely to be wholly or partly familiar with the guidelines than non-psychological specialists. A range of staff co-ordinated delivery of psychological support including supportive care service managers (21%), psychological services managers (17%), hospice directors or chief executives (12%), or clinical psychologists (14%), while 15% of hospices reported nobody was specifically responsible.

### Adequacy of care

**Fig. 1:**
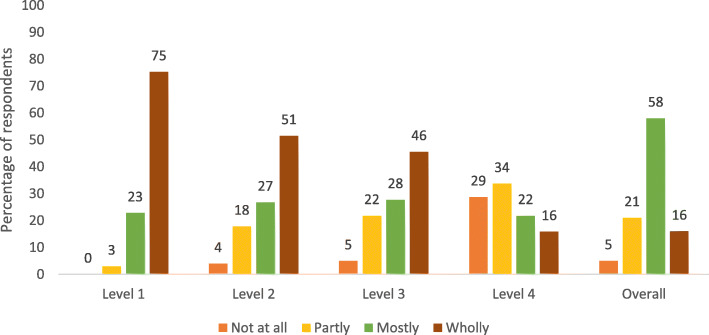
Extent to which patients receive sufficient access to care at each NICE level of psychological support (*n* = 101)

Figure 1 summarises how adequately respondents felt their patients’ needs are being met at each of the NICE levels of psychological care, and overall, on a 4-point scale ranging from wholly met to not at all. Three-quarters (75%) felt patients’ needs were wholly met at Level 1 (basic support). This fell to 51% at Level 2, 46% at Level 3 and just 16% at Level 4 (specialist support) and overall (i.e. across all levels). Hospices in Wales (40%) and Northern Ireland (50%) were more likely than hospices in England (2%) and Scotland (0%) to report that overall care is not at all adequate. The majority of hospices in England (77%), Wales (60%) and Scotland (80%) felt overall care was mostly or wholly adequate, compared to none in Northern Ireland.

### Aspects of psychological support that require improvement and barriers to care: thematic analysis

**Fig. 2:**
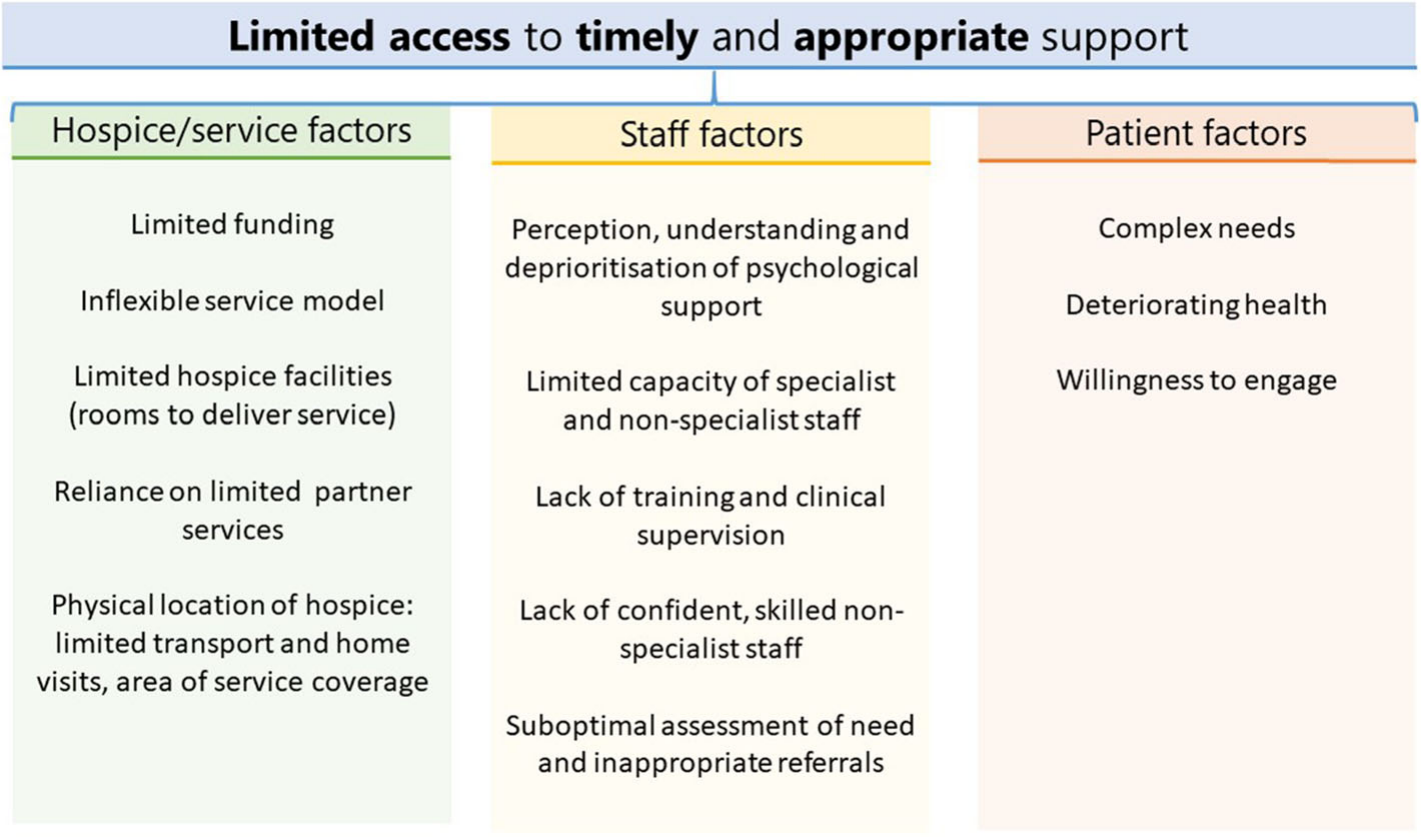
Barriers to providing access to timely and appropriate psychological support in UK hospices

The overarching theme identified was a need for more access to appropriate support in a timely manner. A number of inter-related barriers to care were identified by respondents, which can be classified as hospice, staff or patient factors. These barriers are summarised in Fig. 2.

### Developing best practice and facilitators to care: thematic analysis

This analysis identified the following factors that facilitated the provision of psychological care:

- Having appropriately skilled staff and volunteers available (such as in-house clinical psychologists and counsellors).
- Adequate training and supervision for staff to feel confident providing care at Levels 1 and 2.
- Good communication skills.
- Audit.
- Clear service structure and referral pathways (both internal and external).
- Appropriate assessment of both needs and outcomes of psychological support.
- Committed staff and supportive management who recognise and communicate the importance of psychological support.

## Discussion

Overall, the survey found that there is significant variation in types, organisation and adequacy of psychological support provided across hospices in the UK. Services were managed by a variety of different staff roles. Hospices draw on a range of therapeutic approaches, with mindfulness-based strategies being most widely offered. Nearly all respondents were aware of the NICE four-tier model. In terms of adequacy of care, the majority reported that basic psychological and emotional support (Levels 1 and 2) was mostly or wholly adequate. But just 16% of respondents felt that specialist care for patients with complex psychological needs (Level 4) and overall psychological support were wholly adequate. This pattern is similar to the findings of the last national survey of psychological services in UK hospices from 2005, which found access to psychological care specialists was limited compared to Level 1 and 2 professionals such as complementary therapists and spiritual advisors [13]. Likewise, it supports the findings of the recent survey of psychologists working in end-of-life care settings which found a third of respondents rated their input as ‘not at all sufficient’ [15].

Despite indicating that complex psychological needs are often not fully met for patients in UK hospices, our results suggest a trend towards improved access to specialist psychological support over the last 15 years. In 2005, most hospices did not have access to clinical psychologists (49%), counsellors (62%), psychiatrists (70%) and psychotherapists (90%) respectively. However, our survey found access has improved, with only 19, 5, 20 and 20% of hospices reporting no access to these specialist professionals, respectively. Most hospices had access to at least one of these specialist professionals, with only one hospice reporting that they did not have access to any of these professionals qualified to provide Tier 3 and 4 care. The general trend towards improved access may reflect the increased recognition of the importance of psychological care, both in end-of-life care and for the general population [6, 27].

Despite these improvements, our qualitative analysis revealed some hospices still face complex challenges in fully meeting their patients’ psychological needs. Many noted there was not enough staff capacity to meet patient need, sometimes resulting in long waiting lists. Some felt that, without enough specialist staff available, staff providing care at lower levels did not receive sufficient training and therefore sometimes missed patients that could benefit from referral to higher levels of care. These findings align with those of a recent survey of clinical psychologists which emphasised the ‘missed opportunities’ of referring patients to specialists at an earlier stage [15]. Appropriate needs assessment and referral to higher tiers is highlighted in NICE guidelines as a fundamental principle of the tiered model, and thus could be an important area of focus for hospices developing their psychological services [11]. Future guideline updates should consider providing practical guidance on this (and not just those within a cancer care pathway, as in the current guidelines).

For those struggling with staff capacity, strategies for collaborative working with partner services (e.g., NHS trusts) could be critical. This is particularly pertinent given our findings, in line with other studies, that hospices rely heavily on referral to external services to provide psychological support [13–15], and in light of the ongoing funding challenges facing the hospice sector that may preclude investment in hiring or training internal staff [28]. This recommendation is in line with the findings of a recent scoping review of palliative care for people with severe mental illness, which found that whilst access to care is limited, there is potential for relationships between mental health and palliative care services [29].

Funding for hospice and other end-of-life care services poses a challenge in many countries [9, 30], and as such, the importance of building partnerships has international relevance for services that may be facing similar capacity issues. In the United States, a 2014 survey found that palliative care teams were more likely to report that their patients’ mental health needs were being met if they were with working with an identified psychiatrist, but that co-involvement of teams was limited [31]. Researchers in Australia have also emphasised the importance of collaborations between palliative services and mental health specialists to meet mental health needs at end-of-life, and highlighted a paucity of research in the area [32]. In Singapore, a case-series review study found working with a specialist psychiatrist could improve patients’ well-being and enhance existing home-based hospice care [33]. Also of note is a recent, successful collaboration between psychiatry and palliative care services within a medical centre in the United States, who formed a liaison team to meet a growing need for palliative care, including psychosocial support, during the COVID-19 pandemic [34].

In our survey, when asked to describe facilitators of good care, audit was highlighted. Audit is advocated by national bodies as an important aspect of delivering and maintaining high quality care [35, 36]. We recommend it would be beneficial for those hospices who have not done so already to evaluate the psychological services they provide to inform development of a clear improvement strategy, including, for example, where partnerships with external mental health services may be beneficial. Guidelines for conducting local clinical audits are available from the Healthcare Quality Improvement Partnership [37].

### Strengths and limitations

The response rate is higher than other recent surveys of psychological services in hospice and palliative care settings [14, 15], although not as high as the 74% achieved in the 2005 UK hospice survey [13]. To help maintain response rate, participants could skip questions they did not know the answer to, thus a number of participants skipped at least some questions (particularly the more complex ‘matrix’ style questions), limiting the validity of results. As with all surveys, response bias is an intrinsic risk [38]. Our results may reflect the views of hospices and hospice staff with an interest in psychological care, or with more time and resource. That said, overall the sample was fairly representative of the Hospice UK membership list in terms of location and service management. The sample size was large enough to gather a wide range of views and experiences, and the majority of hospices offered a community service, which is how the majority of hospice care is delivered in the UK [26]. This survey does not capture the views and experiences of people receiving care from hospices. Future research could explore to what extent patient experiences align with staff perceptions, to ensure the recommendations made in this paper speak to a patient-centred care model.

This survey was conducted before the COVID-19 pandemic, which is damaging the funding available to the hospice sector, and changing ways of delivering care [17]. An update and comparison to this survey is recommended following this health crisis as it is likely to significantly impact on the way hospices provide psychological support to their patients, including adaptations to deliver care remotely [39]. Indeed this survey could act as a benchmark for the impact of COVID-19 on psychological services provided by UK hospices. Likewise, this survey could provide key grounding information for any international comparison of psychological services (similar to past work by taskforces set up by the European Association for Palliative Care on carers and spirituality [40–42]) to inform information sharing, standardisation of good practice and guideline development.

### What this study adds

This is an update and extension of existing research into the provision of psychological care in UK hospices [13]. The results provide insights into the psychological services that are currently available in UK hospices, as well as what hospice staff view as key gaps, facilitators and barriers to providing high quality care across the full spectrum of NICE-defined levels.

## Conclusion

The survey indicates that in the UK, access to specialist psychological support has improved over the past decade. Audits, clear referral structures, partnering with external services, and effective assessment of needs and outcomes of psychological support were highlighted as key facilitators for good psychological care. Despite these improvements, a notable proportion of hospices reported that they were unable to fully meet patient needs, with resource and funding identified as common barriers. Developing collaborative partnerships with external services to provide support could be a practical way of addressing these challenges, and is likely to be key on an international level. Future research should also include international surveys and comparisons of psychological services offered by hospices outside the UK to provide valuable global perspectives and context to these results. It will also be important to explore the evolving impact of the ongoing COVID-19 pandemic on the adequacy of psychological care in hospices, and this survey can act as a benchmark.

## Supplementary Information

**Additional file 1.** CHERRIES guidelines_v1_07 Dec 2020.pdf. Completed checklist in line with the CHERRIES guidelines for reporting electronic survey research.

**Additional file 2.** Invitation to participants_v1_07 Dec 2020.pdf. Plain-text version of the invitation email sent to potential participants.

**Additional file 3.** Questionnaire_v1_07 Dec 2020. pdf. A plain-text version of the online questionnaire.

## Data Availability

Anonymous data and supporting metadata will be uploaded to open access repository (ReShare) at the end of the PhD project of which this research forms part (estimated October 2021). Until then, the datasets used and/or analysed during the current study are available from the corresponding author on reasonable request. The following Additional Files are also available online with this manuscript:

## Abbreviations

CBT: Cognitive Behavioural Therapy
CHERRIES: Checklist for Reporting Results of Internet E-Surveys
HRA: Health Research Authority
IQR: Interquartile Range
NHS: National Health Service
NICE: National Institute for Health and Care Excellence
PPI: Patient and Public Involvement
UK: United Kingdom
