# Supplementary material for: Access to and adequacy of psychological services for adult patients in UK hospices: a national, cross-sectional survey
Source: BMC Palliat Care. 2021 Feb 10;20:31. doi: 10.1186/s12904-021-00724-3 (PMC7874563; doi:10.1186/s12904-021-00724-3)
Supplement: Supplementary file 2 — Additional file 2. Invitation to participants_v1_07 Dec 2020.pdf. Plain-text version of the invitation email sent to potential participants. [file 12904_2021_724_MOESM2_ESM.pdf]

ADDITIONAL FILE 2 TITLE PAGE:

INVITATION TO PARTICIPANTS

***

Supporting manuscript:

Access to and adequacy of psychological services for adult patients in UK hospices: a national, cross-sectional survey

Authors:

Daisy McInnerney[1*], Bridget Candy[1], Patrick Stone[1], Nicola Atkin[2], Joana Johnson[3], Syd Hiskey[4], Nuriye Kupeli[1]

Author affiliations:

1. Marie Curie Palliative Care Research Department, Division of Psychiatry, UCL, London, UK
2. Parkville Integrated Palliative Care Service, Peter MacCallum Cancer Centre, Melbourne, Australia
3. Marie Curie Hospice, Hampstead, London, UK
4. The Oaks Hospital, Colchester, UK

*Corresponding author:

Email: daisy.mcinnerney.18@ucl.ac.uk

Phone: 020 3108 5683

Postal address: Wing B, Floor 6, Marie Curie Palliative Care Research Department, Division of Psychiatry, UCL, Maple House, 149 Tottenham Court Road, London, UK

SUBJECT LINE: Invitation to take part in UCL survey of psychological services in UK hospices

Dear [NAME – if known*],

Re: Survey of psychological services in UK hospices

We would like to invite you to take part in a nationwide survey of psychological services in hospices in the UK. The survey is being run by the Marie Curie Palliative Care Research Department at University College London (UCL).

As you are aware, psychological distress is common in people living with a terminal illness. But evidence suggests that for some people receiving palliative care, including those in hospices, access to psychological support is limited. We are running this national survey to explore how psychological services are provided in hospices across the UK. The results will help us understand more about current delivery of and access to these services and provide important evidence to inform future efforts to develop best practice.

As we understand you are closely involved in the organisation and/or delivery of psychological services, we would like to gather your views on how psychological care is provided at the [HOSPICE NAME]. If you think somebody else at your hospice would be better placed to the complete this survey, we would be very grateful if you could pass this email onto them. **We are only looking for one response from each hospice.**

Please read the information sheet which is available here [insert link] for more details about the questionnaire and your role as a participant in the survey. Please download and save the information sheet so that you can easily refer to it at a later date if you like.

Once you have read the information sheet, **click here** to complete the short online questionnaire (approximately 15-20 minutes) or visit: insert link address

The survey will be open until [INSERT DATE].

In recognition of your time, once you've completed the survey you can enter a prize draw to win one of two £30 Amazon gift vouchers. You can also register to receive a certificate acknowledging your contribution to this research.

If you have any questions, please do not hesitate to get in touch with a member of the investigation team - our contact details are provided in the email signatures below.

Many thanks for your interest and participation,

Kind regards,

Miss Daisy McInnerney daisy.mcinnerney.18@ucl.ac.uk

MPhil/PhD student

Marie Curie Palliative Care Research Department, University College London

Dr Bridget Candy b.candy@ucl.ac.uk

Principal Research Associate

Marie Curie Palliative Care Research Department, University College London

Dr Nuriye Kupeli n.kupeli@ucl.ac.uk

Senior Research Associate

Marie Curie Palliative Care Research Department, University College London

Professor Patrick Stone p.stone@ucl.ac.uk

Consultant in Palliative Medicine and Professor of Palliative and End-of-Life Care

Marie Curie Palliative Care Research Department, University College London

*NB/ the name will only be included if this is passed on to us by the person we speak to during initial telephone contact made via publicly available contact details
