# Supplementary material for: Access to and adequacy of psychological services for adult patients in UK hospices: a national, cross-sectional survey
Source: BMC Palliat Care. 2021 Feb 10;20:31. doi: 10.1186/s12904-021-00724-3 (PMC7874563; doi:10.1186/s12904-021-00724-3)
Supplement: Supplementary file 3 — Additional file 3. Questionnaire_v1_07 Dec 2020. pdf. A plain-text version of the online questionnaire. [file 12904_2021_724_MOESM3_ESM.pdf]

ADDITIONAL FILE 3 TITLE PAGE:

PLAIN TEXT VERSION OF QUESTIONNAIRE

***

Supporting manuscript:

Access to and adequacy of psychological services for adult patients in UK hospices: a national, cross-sectional survey

Authors:

Daisy McInnerney[1*], Bridget Candy[1], Patrick Stone[1], Nicola Atkin[2], Joana Johnson[3], Syd Hiskey[4], Nuriye Kupeli[1]

Author affiliations:

1. Marie Curie Palliative Care Research Department, Division of Psychiatry, UCL, London, UK
2. Parkville Integrated Palliative Care Service, Peter MacCallum Cancer Centre, Melbourne, Australia
3. Marie Curie Hospice, Hampstead, London, UK
4. The Oaks Hospital, Colchester, UK

*Corresponding author:

Email: daisy.mcinnerney.18@ucl.ac.uk

Phone: 020 3108 5683

Postal address: Wing B, Floor 6, Marie Curie Palliative Care Research Department, Division of Psychiatry, UCL, Maple House, 149 Tottenham Court Road, London, UK

PAGE 1:

A survey of psychological services in UK hospices: what exists, what works and what may not?

Welcome!

Thank you for taking part in this survey, which asks about your experiences as a member of staff involved in organising or delivering psychological services in a hospice in the UK.

The aim of the questionnaire is to understand more about how hospices provide psychological care to their patients, as well as to their family carers. We will use the results of the survey to identify potential gaps or inequities in the delivery of and access to psychological support in hospices across the UK. As such, your responses could be extremely valuable in helping to inform future efforts to develop best practice for providing psychological care in hospices.

The project was reviewed and given favourable opinion by the University College London (UCL) Ethics Committee on [insert date] (ref: [insert ref number]) and the NHS Health Research Authority on [insert date] (ref: [insert ref number]). The study Chief Investigator is Professor Paddy Stone, UCL.

[NEXT]

PAGE 2:

Study information

You can download and save the Participant Information Sheet [insert hyperlink] for a detailed description of the study. You have been invited to participate because you have been identified as a member of staff who is involved in organising and/or delivering psychological support services in your local hospice in the UK. If you think that a different person within your organisation may be better suited to completing the survey, please forward the invitation email on to them. We are only looking to collect one response from each hospice in the UK.

What does the study involve?

During this survey you will be asked 23 multi-part questions – most are multiple choice but a few ask for more open-ended answers. For participating in this study, you will be given the option to enter a prize draw to win one of two £30 gift vouchers, and to download a certificate of completion to add to your portfolio to demonstrate engagement in research.

How long should the study take?

The survey should not take more than 20 minutes to complete. Time taken was measured in piloting with a group of care providers, who reviewed the questions to make sure they are as quick and simple to fill out as possible.

What will happen to the results?

Participation is completely voluntary and if you decide not to participate, this will be in no way detrimental to you, personally or professionally. Confidential results will be stored by UCL. No individual or organisation will be identifiable in any subsequent publication of the results. If you have any questions or concerns, please do not hesitate to contact the study

a. Respondents were able to review and change their answers through a back button, and to log in and out of the questionnaire.

lead, Daisy McInnerney (daisy.mcinnerney.18@ucl.ac.uk), or the Chief Investigator Professor Paddy Stone (p.stone@ucl.ac.uk)

Full details of the UCL Data Policy are available here: https://www.ucl.ac.uk/legal-services/privacy/participants-health-and-care-research-privacy-notice

[NEXT]

PAGE 3:

Consent

By providing consent to take part in this questionnaire, you are indicating that you meet the following inclusion criteria:

- are aged 18 or above
- are able to understand English well enough to have understood all the information given to you about the study
- work at a UK hospice (on a voluntary or paid basis)
- and are well positioned to comment on the delivery of psychological services at the hospice where you work.

If you do not meet one or more of these criteria, please do not proceed any further. Please pass the invitation to take part onto another person within your organisation who you think may be better suited to completing the survey.

Please check the boxes below if you are suitable and willing to participate. Please note, these boxes are mandatory and so if you do not wish to consent to these terms, you will not be able to participate.

- I have read the notes written on the previous page and the Participant Information Sheet (v2.0 25th July 2019), and understand what the study involves.

- I understand that if I decide at any time that I no longer wish to take part in this project, I can notify the researchers involved and withdraw immediately (up until the point that my data is anonymised)

- I consent to the processing of my personal information for the purposes of this research study.

- I understand that such information will be treated as strictly confidential and handled in accordance with the provisions of the Data Protection Act 2018

- I agree that my non-personal research data may be used by others for future research. I am assured that the confidentiality of my personal data will be upheld through the removal of identifiers.

- I agree that my information can be anonymously quoted in research outputs.

- I agree that the research project named above has been explained to me to my satisfaction and I agree to take part in this study.

a. Respondents were able to review and change their answers through a back button, and to log in and out of the questionnaire.

Please provide your email address in the box below to signify your consent to these terms.

You can use your email address to log in and out of this questionnaire so that you can return to it and complete it in more than one sitting if needed. We will delete your email address at the end of the study, and will not contact you again, unless you explicitly request for us to do so at the end of this questionnaire.

Email address: FREE TEXT

[CONFIRM AND START QUESTIONNAIRE]

PAGE 4:

Section 1: Basic information

1. What is your job title? Select all that apply.
    - Clinical services manager
    - Chief executive
    - Clinical psychologist
    - Hospice manager
    - Patient and family support services manager
    - Psychological services manager
    - Supportive care services manager
    - Medical doctor
    - Other doctor
    - Other (please specify: FREE TEXT)

2. Where is your hospice?
    - Scotland
    - Northern Ireland
    - Wales
    - North East England
    - North West England
    - Yorkshire and the Humber
    - West Midlands (England)
    - East Midlands (England)
    - South West England
    - South East England
    - East of England
    - Greater London

3. Is your hospice NHS-managed?
    - An independent charity (even if partly funded by the NHS)
    - An NHS hospice (even if partly funded by charity)
    - Other (please specify: FREE TEXT)

4. In your hospice:

a. Respondents were able to review and change their answers through a back button, and to log in and out of the questionnaire.

a. Do you have hospice inpatient beds?
    - Yes
    - No
        - FILTER QUESTIONS – only if answer Yes to 4a
        - How many beds do you have? FREE TEXT
        - Approximately how many admissions per week do you take? *If you are completely unsure, please leave blank.* FREE TEXT

b. Do you have a day-centre?
    - Yes
    - No
        - FILTER QUESTIONS – only if answer Yes to 4b
        - Approximately how many patients per week attend? *If you are completely unsure, please leave blank.* FREE TEXT

c. Do you offer general palliative care outpatient clinics at the hospice?
    - Yes
    - No
        - FILTER QUESTIONS – only if answer Yes to 4c
        - Approximately how many patients per week attend? *If you are completely unsure, please leave blank.* FREE TEXT

d. Do you have a community palliative care team?
    - Yes
    - No
        - FILTER QUESTIONS – only if answer Yes to 4d
        - Approximately how many patients are on your caseload at any one time? *If you are completely unsure, please leave blank.* FREE TEXT
        - Approximately how many new referrals do you receive per week? *If you are completely unsure, please leave blank* FREE TEXT

5. What type of people does your hospice provide care for? Select all that apply.
    - Adults
    - Children (as patients)
    - People with cancer
    - People with life threatening illnesses other than cancer
    - Other (please specify: FREE TEXT)

PAGE 3

Section 2: Organisation of psychological support services at your hospice

6. Which of the following best describes how familiar you are with the National Institute of Clinical Excellence (NICE) model of professional psychological assessment and support (pictured below in Figure 1)?

a. Respondents were able to review and change their answers through a back button, and to log in and out of the questionnaire.

The model was published in their guidelines for palliative care for cancer patients. At the time of this survey, NICE guidelines for psychological care of people receiving palliative care who do not have cancer are not available.

- Not at all familiar (e.g. I have never heard of the guidelines before now)
- Partly (e.g. I have heard of the guidelines, but don't know what they recommend)
- Mostly (e.g. I have heard of the guidelines, and have a general idea of what they recommend)
- Wholly (e.g. I have a good understanding of the model and guideline recommendations)

[image of NICE guidelines table]

Figure 1. Model of professional psychological assessment and support. (Adapted from National Institute for Clinical Excellence. (2004). Guidance on cancer services: improving supportive and palliative care for adults with adults the manual. London: National Institute for Clinical Excellence. The full guidelines are available at the NICE website here.)

7. Who does your hospice offer psychological support services to and at what level is the care provided? The levels are based on the framework described by the NICE recommended model of psychological support shown above. Please select all that apply.

	**Inpatients**	**Day care patients**	**Home care patients**	**Family carers**	**Staff**	**Other (please specify):**<br>- **User**<br>- **Level of support**
**Level 1**						FREE TEXT
**Level 2**						FREE TEXT
**Level 3**						FREE TEXT
**Level 4**						FREE TEXT

8. Who is/are responsible for co-ordinating the delivery of psychological support services at the hospice you work at? Please select all that apply.
   - Supportive care services manager
   - Psychological services manager
   - Hospice Director / Chief Executive
   - Clinical psychologist
   - Nobody is specifically in charge of co-ordinating psychological support services
   - Other (please specify): FREE TEXT

PAGE 4

Section 3: Types of psychological support at your hospice

a. Respondents were able to review and change their answers through a back button, and to log in and out of the questionnaire.

9. Which of the following psychological support professionals does the hospice make available to ***adult patients***? Please indicate:
    A. Whether the service is available in house or by referral to an external service
    B. Whether the service is provided by paid employees or volunteers.

    Please select all options that apply

**A**	**Available in-house**		**Available by referral to an external service**		**Not available**		**Unknown**	
**B**	*Employee*	*Voluntary*	*Employee*	*Voluntary*	*Employee*	*Voluntary*	*Employee*	*Voluntary*
**Spiritual advisor / chaplain**								
**Complementary therapist**								
**Social worker**								
**Creative therapist (e.g. art therapist, music therapist)**								
**Clinical psychologist**								
**Counselling psychologist**								
**Counsellor**								
**Psychiatrist**								
**Dual qualified professional providing psychological support (i.e. an individual with two professional trainings such as someone trained as both a social worker and counsellor)**								
**Registered Mental Health Nurse (RMN)**								
**Psychotherapist**								
**Mental health nurse (RMN)**								
**Occupational therapist**								
**Other (please specify):**<br>• **Role**<br>• **In house/ external**<br>• **Voluntary/paid**	FREE TEXT							

10. If you have a dual-qualified professional available in-house, please can you describe the roles they are dually qualified in? Please also explain whether they perform *both* roles which they are qualified in as part of their job at the hospice (e.g. a dual qualified counsellor and psychotherapist may provide only counselling but not psychotherapy at the hospice, OR they may provide both services)

a. Respondents were able to review and change their answers through a back button, and to log in and out of the questionnaire.

FREE TEXT

11. Which of the following psychological support professionals does the hospice make available to ***family carers***? Please indicate:
    A. Whether the service is available in house or by referral to an external service
    B. Whether the service is provided by paid employees or volunteers.

A	Available in-house		Available by referral to an external service		Not available		Unknown	
B	*Employee*	*Voluntary*	*Employee*	*Voluntary*	*Employee*	*Voluntary*	*Employee*	*Voluntary*
**Spiritual advisor / chaplain**								
**Complementary therapist**								
**Social worker**								
**Creative therapist (e.g. art therapist, music therapist)**								
**Clinical psychologist**								
**Counselling psychologist**								
**Counsellor**								
**Psychiatrist**								
**Dual qualified professional (i.e. an individual with two professional trainings such as someone trained as both a social worker and counsellor)**								
**Registered Mental Health Nurse (RMN)**								
**Psychotherapist**								
**Mental health nurse (RMN)**								
**Occupational therapist**								
**Other (please specify):**<br>• **Role**<br>• **In house/ external**<br>• **Voluntary/paid**	FREE TEXT							

12. Which of the following psychological therapies are drawn on at your hospice as part of the support offered to:
    A. ***adult patients***
    B. ***family carers***

    If yes, please indicate the job role / level of the person who delivers the therapy (e.g. psychotherapist, clinical psychologist)

a. Respondents were able to review and change their answers through a back button, and to log in and out of the questionnaire.

**Therapy name**	**A. Adult patients**				**B. Family carers**			
	Yes	No	Don't know		Yes	No	Don't know	If yes, who by?
**Cognitive Behavioural Therapy**								FREE TEXT
**Acceptance and Commitment Therapy**								FREE TEXT
**Compassion Focused Therapy**								FREE TEXT
**Mindfulness Strategies**								FREE TEXT
**Narrative Therapy**								FREE TEXT
**Solution Focused Therapy**								FREE TEXT
**Systemic Therapy**								FREE TEXT
**Psychodynamic approaches**								FREE TEXT
**Psychotherapeutic approaches**								FREE TEXT
**Music therapy**								FREE TEXT
**Art therapy**								FREE TEXT
**Writing-based therapy**								FREE TEXT
**Hypnotherapy**								FREE TEXT
**Other (please specify):**<br>• Type of therapy<br>• Who delivered by (role)	FREE TEXT FREE TEXT FREE TEXT				FREE TEXT FREE TEXT FREE TEXT			

PAGE 5

Section 4: Access to psychological support at your hospice

13. Based on the framework described by the NICE recommended model of psychological support, to what extent do you believe ***adult patients and family carers*** at your hospice have sufficient access to Level 1 support?

	**Wholly**	**Mostly**	**Partly**	**Not at all**
**Adult patients**				
**Family carers**				

14. To what extent do you believe ***adult patients and family carers*** at your hospice have sufficient access to Level 2 support?

	**Wholly**	**Partly**	**Partly**	**Not at all**
**Adult patients**				
**Family carers**				

15. To what extent do you believe ***adult patients and family carers*** at your hospice have sufficient access to Level 3 support?

a. Respondents were able to review and change their answers through a back button, and to log in and out of the questionnaire.

	Wholly	Partly	Partly	Not at all
**Adult patients**				
**Family carers**				

16. To what extent do you believe ***adult patients and family carers*** at your hospice have sufficient access to Level 4 support?

	Wholly	Partly	Partly	Not at all
**Adult patients**				
**Family carers**				

17. Do you believe the overall provision of psychological support currently provided to ***adult patients and family carers*** attending your hospice is sufficient?

	Wholly	Partly	Partly	Not at all
**Adult patients**				
**Family carers**				

18. In your opinion, are there any particular aspects of psychological care provision at your hospice that you feel could be improved for ***adult patients***?
    - Yes
    - No
        - FILTER QUESTIONS – only if answer Yes to 18
        - Please specify which aspects of psychological care provision you feel could be improved?

19. In your opinion, are there any particular aspects of psychological care provision at your hospice that you feel could be improved for ***family carers***?
    - Yes
    - No
        1. FILTER QUESTIONS – only if answer Yes to 20
        2. Please specify which aspects of psychological care provision you feel could be improved?

20. In your opinion, what are the main barriers to providing effective psychological care to ***adult patients*** in your hospice (if any)?
    FREE TEXT

21. In your opinion, what are the main barriers to providing effective psychological care to ***family carers*** in your hospice (if any)?
    FREE TEXT

22. In your opinion, what are the main facilitators to providing effective psychological care to ***adult patients*** in your hospice?
    FREE TEXT

a. Respondents were able to review and change their answers through a back button, and to log in and out of the questionnaire.

23. In your opinion, what are the main facilitators to providing effective psychological care to ***family carers*** in your hospice?
    FREE TEXT

PAGE 6

Thank you for taking part!

You've finished! Thank you very much for taking the time and thought to contribute to this survey.

If you have any further comments you would like to make about the psychological services provided in your hospice, please write them below:

FREE TEXT

What next?

A. Would you like to receive an email with the results of the survey and/or any resulting publications?

- Yes
- No

B. Would you be interested in hearing more about potential opportunities to take part in future research studies with this research team on the topic of psychological support in palliative care?

This research will be run as part of an ESRC and Marie Curie co-funded PhD project looking into developing an emotional disclosure-based psychotherapeutic intervention for use in palliative care. It may be in the form of further questionnaires, focus groups, interviews or co-design workshops. By providing your contact details here, you are giving your permission for us to securely store your contact details for the duration of the PhD project (until October 2021). Whilst we may use these contact details to invite you to take part in future research, you will be under no obligation to respond or participate. Your contact details will not be linked to your survey responses.

- Yes
- No

C. Would you like to enter the prize draw to win one of two £30 vouchers?

- Yes
- No

D. Would you like to receive a personalised certificate recognising your completion of this survey?

We will email you the personalised certificate within 2 weeks of the closing date of the survey.

- Yes
- No

a. Respondents were able to review and change their answers through a back button, and to log in and out of the questionnaire.

- FILTER QUESTIONS – if answered yes to question C
- Please provide your name to be included on the certificate. Please spell this exactly as you would like it to appear. These details will not be linked to your survey responses: FREE TEXT

If you have any questions about the study please let one of the investigators know by email (see contact details below).

Research team contact details

Ms Daisy McInnerney daisy.mcinnerney.18@ucl.ac.uk

Dr Bridget Candy b.candy@ucl.ac.uk

Dr Nuriye Kupeli n.kupeli@ucl.ac.uk

Prof. Patrick Stone p.stone@ucl.ac.uk

a. Respondents were able to review and change their answers through a back button, and to log in and out of the questionnaire.
